# Arsenic Trioxide Decreases Lymphangiogenesis by Inducing Apoptotic Pathways and Inhibition of Important Endothelial Cell Receptors

**DOI:** 10.3390/cimb46010006

**Published:** 2023-12-21

**Authors:** Igor Hrgovic, Eva Zöller, Monika Doll, Tsige Hailemariam-Jahn, Thilo Jakob, Roland Kaufmann, Markus Meissner, Johannes Kleemann

**Affiliations:** 1 Department of Dermatology and Allergy, Experimental Dermatology and Allergy Research Group, University Medical Center Giessen, Justus Liebig University, 35392 Giessen, Germany; 2 Department of Dermatology, Venereology and Allergy, Goethe University, 60596 Frankfurt am Main, Germany

**Keywords:** arsenic trioxide (ATO), lymphangiogenesis, apoptotic pathways, Tie-2, VEGFR-2/3, Lyve-1

## Abstract

Tumor-induced lymphangiogenesis is strongly associated with the formation of tumor metastasis. Therefore, the regulation of lymphangiogenesis offers a promising target in cancer therapy. Arsenic trioxide (ATO) is highly effective in the treatment of patients with acute promyelocytic leukemia (APL). As ATO mediates anti-angiogenic effects on endothelial and tumor cells, we aimed to explore the impact of ATO on lymphangiogenesis in human lymphatic endothelial cells (LEC). The BrdU assay and flow cytometry analysis were used to evaluate the influence of ATO on the proliferation and cell cycle distribution of LECs. The lymphatic suppression effects of ATO were investigated in vitro using the lymphatic tube formation assay. The effects of ATO on apoptosis, mitochondrial membrane potential and endothelial cell receptors were investigated by Western blotting, ELISA, flow cytometry and qRT-PCR. The treatment of LECs with ATO attenuated cell proliferation, blocked tube formation and induced subG0/G1 arrest in LECs, thus suggesting enhanced apoptosis. Although subG0/G1 arrest was accompanied by the upregulation of p21 and p53, ATO treatment did not lead to visible cell cycle arrest in LECs. In addition, ATO caused apoptosis via the release of cytochrome c from mitochondria, activating caspases 3, 8 and 9; downregulating the anti-apoptotic proteins survivin, XIAP and cIAP-2; and upregulating the pro-apoptotic protein Fas. Furthermore, we observed that ATO inhibited the VEGF-induced proliferation of LECs, indicating that pro-survival VEGF/VEGFR signaling was affected by ATO treatment. Finally, we found that ATO inhibited the expression of the important endothelial cell receptors VEGFR-2, VEGFR-3, Tie-2 and Lyve-1. In conclusion, we demonstrate that ATO inhibits lymphangiogenesis by activating apoptotic pathways and inhibiting important endothelial cell receptors, which suggests that this drug should be further evaluated in the treatment of tumor-associated lymphangiogenesis.

## 1. Introduction

Various derivatives of arsenic have been used for more than 2000 years as a therapeutic drug for many diseases, such as malaria, syphilis and the plague [1]. Arsenic trioxide (ATO) is an inorganic compound with the formula As_2_O_3_. Despite the toxicity of arsenic compounds, ATO has been used in a number of Chinese and Indian medicines to treat cancer [2]. In the late 1990s, interest in ATO treatment options for acute promyelocytic leukemia (APL) widely emerged. In 1998, Soignet and colleagues showed the first successful treatment outcomes of APL with arsenic trioxide in the USA [3]. Since then, several studies have confirmed the efficacy of ATO in the treatment of APL [4]. Currently, combined ATO and all-trans retinoic acid (ATRA) is strongly recommended as a first-line therapy in the treatment of low- and intermediate-risk APL, with complete remission rates and 4-year survival rates of more than 90% [5,6]. Although the toxicity of arsenic compounds can vary greatly, ATO has demonstrated a favorable safety profile in patients with APL [7]. In the context of treating APL, ATO induces terminal differentiation both in vitro and in vivo via the proteasomal degradation of the retinoic acid receptor-alpha gene (PML-RARα) fusion protein, which is mainly involved in the pathogenesis of APL [8]. ATO induces differentiation, growth arrest or apoptosis in a number of tumor cell lines in vitro. In addition, ATO has been shown to suppress anti-apoptotic genes (e.g., survivin, bcl-2 and Mcl-1) and to upregulate pro-apoptotic genes (e.g., Puma, Bax and NOXA) in various tumor cell lines, such as leukemia, glioma and rhabdomyosarcoma [9,10,11]. Furthermore, ATO regulates the expression of proteins implicated in cell cycle control and differentiation (e.g., p21^WAF1/KIP1^, p53, E2F1, HoxC9, cyclin E and D) [12,13,14]. Moreover, ATO has been reported to suppress angiogenesis by inhibiting pro-angiogenic factors, such as vascular endothelial growth factor (VEGF), Notch1 and VEGFR-2 [15,16]. Lymphangiogenesis is the formation of new lymphatic vessels from preexisting ones. In adults, lymphangiogenesis mainly occurs during the development of the corpus luteum, tissue repair, wound healing or regeneration [17]. Thus far, several studies have revealed the importance of lymphangiogenesis for the formation of metastasis in the tumor-draining lymph nodes. Due to this fact, increased tumor-related lymphangiogenesis correlates with the formation of metastasis and a poor prognosis in patients with breast cancer and melanoma [18]. Thus, targeting tumor lymphangiogenesis may provide a promising approach to treat cancer metastasis. The first evidence in a gastric cancer model revealed that As_2_O_3_ may inhibit lymphangiogenesis by suppressing the expression of VEGF-C and VEGFR-3 in gastric cancer cells [19]. Based on these findings, we investigated the anti-lymphangiogenic effects of ATO in primary human lymphatic endothelial cells.

## 2. Materials and Methods

### 2.1. Cell Culture

Cryopreserved primary human dermal lymphatic endothelial cells were obtained from PromoCell (Heidelberg, Germany). The cells were isolated from the dermis of juvenile foreskin from single donors. LECs were cultured at 37 °C and 5% CO_2_ in an endothelial cell basal medium MV2 (PromoCell, Heidelberg, Germany) supplemented with 7.5% human serum (Corning, Corning, NY, USA). Cells at passages 4–8 were used in this study.

### 2.2. Chemicals

Arsenic trioxide (ATO) was purchased from TEVA Pharma (Ulm, Germany), staurosporine from Sigma-Aldrich (Taufkirchen, Germany) and cytochalasin D from Biomol (Hamburg, Germany). Recombinant human VEGF-A and VEGF-C were obtained from ReliaTech (Wolfenbüttel, Germany).

### 2.3. Fluorescence-Activated Cell Sorting Analysis 

LECs were blocked in the S-phase of the cell cycle by serum deprivation for 24 h. The cells were then treated with 5 µM ATO or 1 µM staurosporine as a control for 24 h. After the treatment, cells were washed once with 1× PBS, trypsinized and washed again with PBS. The cells were fixed in ice-cold 70% ethanol for 30 min and 4 °C. After fixation of the cells and subsequent centrifugation, cell pellets were incubated in PBS containing 40 µg/mL RNase A for 30 min at 37 °C and resuspended in PBS containing 50 µg/mL propidium iodide. Cell cycle analysis was performed using a BD Accuri™ C6 flow cytometer (Becton Dickinson, Franklin Lakes, NJ, USA).

### 2.4. Cell Proliferation and Cytotoxicity Assay

The impact of ATO on cell proliferation was determined using the ELISA-BrdU from Roche Diagnostics (Mannheim, Germany). The detailed cell assay processes were performed as described previously [20]. The effect of ATO on cytotoxicity in LECs was tested using the cytotoxicity detection kit (Roche Diagnostics, Mannheim, Germany), measuring lactate dehydrogenase (LDH) released from the cells in the supernatant. For this assay, cells were seeded in triplicate into 96-well plates (1 × 10^5^/well) and allowed to adhere overnight. Then, they were incubated with increasing concentrations of ATO (0.5–10 µM) or 0.1% Triton-X100 (Sigma-Aldrich, Taufkirchen, Germany) as a positive control for 24 h. Then, 50 µL of the supernatant was transferred into empty wells of a 96-well plate, mixed with 50 µL of the provided LDH reaction mix and incubated in the dark for 30 min at RT. To quantify LDH release, absorbance measurements at 500 nm were conducted using the photometer ASYS Expert96 (Deelux Labortechnik, Gödenstorf, Germany). The data from each treatment group are expressed as a percentage of the control group.

### 2.5. Tube Formation Assay

LECs were treated with increasing concentrations of ATO (0.5–10 µM) or 6 µM cytochalasin D as a positive control for 24 h in endothelial cell basal medium MV2 supplemented with 7.5% human serum, without the addition of the growth factors VEGF and bFGF from the supplemental mix. Growth factor-reduced Matrigel^TM^ (BD Discovery Labware, Bedford, MA, USA) supplemented with 0.5 ng/mL VEGF (recombinant human VEGF 165) and 10 ng/mL FGF was placed into the lower chambers of µ-slide angiogenesis wells (IBIDI, Planegg/Martinsried, Germany) and allowed to polymerize for 30 min at 37 °C. Then, treated LECs were resuspended in growth media supplemented with VEGF and FGF and seeded on the gel in duplicate at a density of 1 × 10^4^ cells/well. Cytochalasin D is a mycotoxin and acts as a positive control in this assay due to its ability to inhibit tube formation. Four pictures per well were taken after 6 h using a 200× magnification in phase contrast mode. Images were taken on the Biozero BZ-8000K microscope (Keyence Deutschland GmbH, Frankfurt am Main, Germany). Images were acquired via the BZ analyzer software 2.1 (Keyence Deutschland GmbH, Frankfurt am Main, Germany). Using the tool Angiogenesis Analyzer [21] in the software ImageJ v1.52a (U.S. National Institutes of Health, Bethesda, MD, USA), the tube formation ability was analyzed by counting the master segments of each picture.

### 2.6. Apoptosis Assay

The effect of ATO on apoptosis was assessed by measuring cytoplasmic histone-associated DNA fragments (mono- and oligonucleosomes) after induced cell death using the Cell Death Detection ELISA PLUS Kit from Roche Diagnostics (Mannheim, Germany). The detailed cell assay processes were performed as described previously [20].

### 2.7. Caspase Assays

To analyze the ability of ATO to activate caspases 3/7, 8 and 9, Caspase-Glo^®^ Assays were conducted (Promega, Madison, WI, USA). LECs were seeded in triplicate into black 96-well plates at a density of 1.5 × 10^4^ cells per well. Twenty-four hours later, the cells were treated with increasing concentrations of ATO (2.5 µM, 5 µM and 10 µM). After 24 h, the medium was removed and the caspase 3/7, 8 or 9 activity was determined using a luminescent Caspase-Glo^®^ 3/7, 8 or 9 assay kit according to the manufacturer’s protocol. The luminescence was measured using a CytoFluor^®^ Series 4000 (Applied Biosystem, Waltham, MA, USA).

### 2.8. Cytochrome c Release

LECs were plated in cell culture dishes (5 × 10^6^ cells/60 cm^2^) and treated the next day with ATO at concentrations of 2.5, 5 and 10 µM. After 24 h, cytoplasmic extracts were obtained by digitonin permeabilization, as described previously [20]. To analyze the cytochrome c levels in cytosol, an enzyme-linked immunosorbent assay (R&D Systems, Wiesbaden, Germany) was conducted according to the manufacturer’s manual. Optical density (450 nm) was measured using an ELISA reader (ELISA Reader ASYS Expert 96, Deelux Labortechnik, Gödenstorf, Germany).

### 2.9. Western Blot Analysis

Protein samples were prepared as described previously [22]. Following SDS-PAGE and electroblotting, membranes were incubated with the following primary antibodies: anti-p21 (diluted 1:1000) (#2947, Cell Signaling, Danvers, MA, USA), anti-p53 (diluted 1:1000) (#9282, Cell Signaling, Danvers, MA, USA), anti-cIAP-1 (diluted 1:1000) (#4952, Cell Signaling, Danvers, MA, USA), anti-cIAP-2 (diluted 1:1000) (#3130, Cell Signaling, Danvers, MA, USA), anti-survivin (diluted 1:1000) (#2803, Cell Signaling, Danvers, MA, USA), anti-XIAP (diluted 1:1000) (#2042, Cell Signaling, Danvers, MA, USA), anti-Fas (dilutes 1:1000) (#4233, Cell Signaling, Danvers, MA, USA), anti-tubulin (diluted 1:2000) (#3873, Cell Signaling, Danvers, MA, USA), anti-Lyve-1 (diluted 1:500) (FAB20891, R&D Systems, Minneapolis, MN, USA), anti-Tie2 (diluted 1:1000) (sc-324, Santa Cruz, CA, USA), anti-VEGFR-2 (diluted 1:1000) (#9698, Cell Signaling, Danvers, MA, USA) and anti-VEGFR-3 (diluted 1:500) (sc-321, Santa Cruz, CA, USA). Primary antibody application was followed by incubation with horseradish peroxidase-conjugated secondary antibodies (anti-mouse and anti-rabbit IgG, Amersham, Uppsala, Sweden; anti-goat, Dako, Glostrup, Denmark). Western blot analysis was performed as described earlier [20]. Protein bands were quantified by using ImageJ (v1.52a). Optical band densities were normalized with the corresponding tubulin bands.

### 2.10. Real-Time Quantitative PCR Analysis

To analyze the gene expression of VEGFR-3 and Lyve-1 on the RNA level, total cellular RNA extraction was conducted using QIAshredder columns (Qiagen, Hilden, Germany) and an RNeasy Mini Kit (Qiagen, Hilden, Germany) as recommended by the manufacturer after DNase digestion. First, 750 ng of RNA was used for first-strand cDNA synthesis using the QuantiTect RT-Kit (Qiagen, Hilden, Germany). Measurements of the relative mRNA amounts of the target genes were conducted using the SYBRgreen dye technique on a LightCycler system (Roche Diagnostics, Mannheim, Germany). Ct values (cycle at which the SYBR Green intensity exceeds a threshold) were measured by using the LightCycler Software Version 4.05 (Roche). The relative gene expression rates were calculated using the 2^−ΔΔCt^ method [23] with normalization to the housekeeping gene β-actin.

### 2.11. Mitochondrial Membrane Potential Measurements

To investigate the influence of ATO on mitochondrial membrane potential (∆ψm), the tetramethylrhodamine ethyl ester perchlorate (TMRE) mitochondrial membrane potential assay was used (Abcam, Cambridge, UK). Human lymphatic endothelial cells were seeded into 6-well plates and allowed to adhere overnight. The next day, the cells were treated with increasing concentrations of ATO (0.5–10 µM) for 6 h or 100 nM carbonyl cyanide 4-(trifluoromethoxy)phenylhydrazone (FCCP) for 10 min. FCCP was used as a positive control due to its ability to uncouple ionophores and so to eliminate mitochondrial membrane potential. After the treatment, the medium was removed from the cells and the TMRE working solution (100 nM TMRE in 1x PBS + 0.2% BSA) was transferred to the cells. To stain the cells, they were incubated for 20 min at 37 °C and 5% CO_2_. During the incubation, TMRE, a red-orange dye, was transported inside the cells through the membrane and accumulated in the negatively charged mitochondria due to its positive charge. In the event of decreased membrane potential, mitochondria are less negative, which leads to decreased fluorescent intensity due to lower concentrations of TMRE at the mitochondria. After 20 min, cells were trypsinized from the plate. After washing the pellets two times with 1x PBS, they were resuspended in 150 μL 1x PBS for FACS measurement. Fluorescent intensities were measured using a flow cytometer. After gating for cell populations, the FL-2 intensities (excitation: 488 nm; emission max.: 575 nm) were compared using the BD Accuri™ C6 software.

### 2.12. Intracellular ROS Measurement

The production of intracellular reactive oxygen species (ROS) induced by ATO was assessed using the 2′,7′-dichlorofluorescin diacetate (DCFDA) Cellular ROS Detection Assay Kit (Abcam, Cambridge, UK). LECs were seeded in triplicate into black 96-well plates (1.5 × 10^4^) and allowed to adhere overnight. After washing the cells with the provided 1x buffer, they were stained with 25 μM DCFDA for 45 min at 37 °C and 5% CO_2_. After a second washing step, the cells were treated with increasing concentrations of ATO (0.5–10 µM) and 50 μM tert-butyl hydrogen peroxide (TBHP) as a positive control for 24 h at 37 °C and 5% CO_2_. During the incubation, the non-fluorescent substrate in the cells was oxidized by ROS into the highly fluorescent compound 2′,7′-dichlorofluorescein (DCF). Unstained cells were included to eliminate background fluorescence. After 24 h, the fluorescence intensity was immediately measured in a microplate reader (CytoFluor 4000, Applied Biosystems, Waltham, MA, USA) with λexc 495 nm and λem 529 nm.

### 2.13. Statistical Analysis

If not stated otherwise, experiments were carried out at least three times independently. Cytotoxicity, proliferation, cytochrome c, ROS and caspase activity assays were carried out at least in triplicate per experiment. Data analysis was performed using the SigmaPlot 12.0 software (Systat Software Inc., San Jose, CA, USA) and data were expressed as means ± standard deviation (SD). The normal distribution of the data was assessed using the Shapiro–Wilk normality test. Subsequently, for differences between more than two groups, the Kruskal–Wallis test was applied, followed by Dunn‘s post hoc analysis. The Mann–Whitney rank sum test was applied to determine differences between two groups. A *p* value of less than 0.05 was considered statistically significant.

## 3. Results

The effects of ATO on cell toxicity, cell proliferation and in vitro lymphangiogenesis in human lymphatic endothelial cells were evaluated.

**Figure 1:**
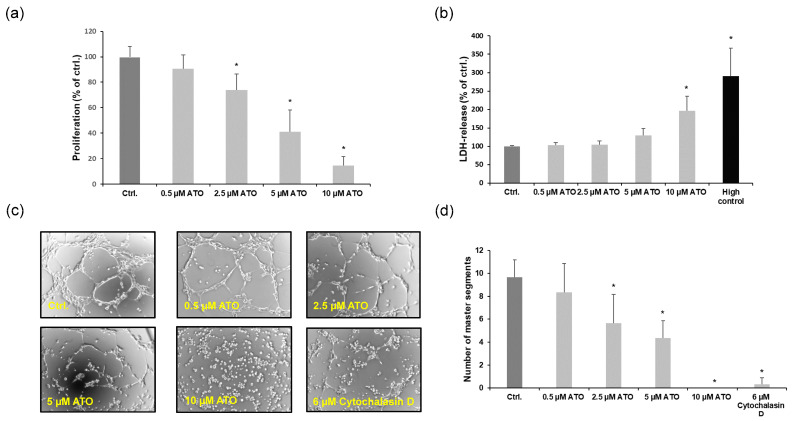
Effects of ATO on the proliferation and cytotoxicity in LECs. Cells were treated with ATO (0.5, 2.5, 5 and 10 µM) for 24 h as indicated. (**a**) The proliferation of LECs was analyzed by BrdU assay. The data are represented as the mean ± SD, each obtained in triplicate from at least six independent experiments. * *p* < 0.05 vs. ctrl. (**b**) Cytotoxicity was examined at 24 h by LDH assay. Values are represented as the mean ± SD, each assessed in triplicate (*n* = 6). Significance was determined by Kruskal–Wallis test with * *p* < 0.05 vs. ctrl. (**c**) Tube formation assay was conducted with LECs treated with increasing concentrations of ATO (0.5–10 µM) and with a positive control (6 µM cytochalasin D) for 24 h; 200× magnification, phase contrast. (**d**) Mean values of master segments analyzed with the tool “Angiogenesis Analyzer for ImageJ” (v1.52a) from three independent experiments are shown as mean ± SD. Significance was determined by Kruskal–Wallis test with * *p* < 0.05 vs. ctrl.

First, we examined the effects of ATO on cell proliferation and cytotoxicity using LECs. In our experiments, we found that ATO reduced cell proliferation in a dose-dependent manner, as determined by the BrdU assay (Figure 1a). To assess the cytotoxic effects of ATO, we performed lactate dehydrogenase assays and found that treating LECs with 0.5, 2.5 or 5 µM ATO for 24 h did not lead to significant changes in LDH release compared to the untreated control, whereas ATO at a concentration of 10 µM led to a significant increase in detected LDH (Figure 1b). Additionally, we found that ATO inhibited capillary-like structure formation in a concentration-dependent manner (Figure 1c,d). These findings provide evidence that ATO mediates anti-lymphangiogenic effects by influencing endothelial cell function.

### 3.1. ATO Induces Apoptotic Cell Death in LECs

**Figure 2:**
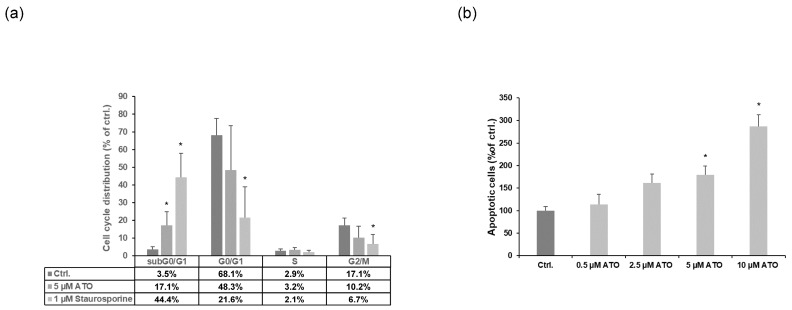
ATO induces apoptosis in LECs. (**a**) LECs were treated with 5 µM of ATO, 1 µM staurosporine as positive control and H_2_O/DMSO as solvent control for 24 h (*n* = 6). Cell cycle distribution was analyzed by flow cytometry after propidium iodide staining. The figure shows the percentage of cells in each phase of cell cycle with regard to solvent controls (=100%; H_2_O/DMSO). Significance was determined by Mann–Whitney rank sum test with * *p* < 0.05 vs. ctrl. (**b**) Cells were treated with 0.5, 2.5, 5 and 10 µM ATO for 24 h as indicated. ATO-induced apoptosis was measured by using a histone/DNA apoptosis ELISA assay. The means ± SD of the six independent experiments performed in triplicate are shown. Significance was determined by Kruskal–Wallis test with * *p* < 0.05 vs. ctrl.

Given the effect of ATO on LEC proliferation, we next monitored the cell cycle phase distribution by flow cytometry. Our data revealed that the percentage of cells in the subG0/G1 phase, which contains apoptotic cells, was significantly increased upon ATO treatment for 24 h (Ctrl.: 3.5 ± 1.5%; 5 µM ATO: 17.1 ± 7.8%). The staurosporine control exhibited 44.4% of cells in the subG0/G1 phase (Figure 2a). Furthermore, we found that ATO did not influence the other phases of the cell cycle, and only staurosporine treatment led to a significant decrease in cells in the G0/G1 and G2/M phases. These findings were further verified by using a histone/DNA ELISA. As shown in Figure 2b, we found that the 24 h incubation of LECs with 5 and 10 µM ATO significantly induced apoptosis. In summary, these findings indicate that ATO leads to an increase in apoptosis but not to an arrest in other cell cycle phases.

### 3.2. ATO Activates Both the Intrinsic and Extrinsic Apoptotic Pathways in Human Lymphatic Endothelial Cells

**Figure 3:**
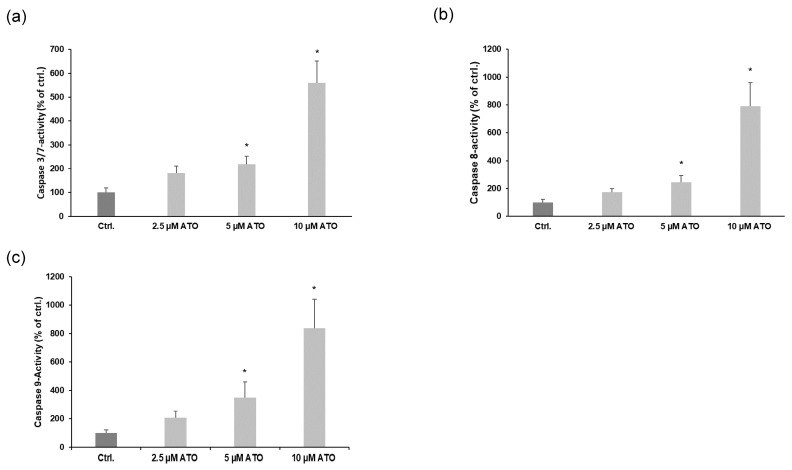
ATO treatment induces both the intrinsic and extrinsic apoptotic pathways in LECs. (**a**–**c**) Quantification of caspase 3/7, 8 and 9 activity in LECs after application with increasing concentrations of ATO (2.5–10 µM) for 24 h. Data are expressed as means ± SD from five independent triplicate experiments. Significance was determined by Kruskal–Wallis test with * *p* < 0.05 vs. ctrl.

To further determine the underlying mechanisms of ATO-mediated LEC apoptosis, we explored whether ATO-induced apoptosis was related to caspase activation. As shown in Figure 3a–c, we found that ATO increased the activation of caspases 3/7, 8 and 9 after 24 h exposure in a dose-dependent manner. These results reveal that ATO induces LEC apoptosis through both extrinsic and intrinsic signaling pathways.

### 3.3. ATO Triggers Apoptosis through Mitochondrial Pathway but Is Independent of Induction of ROS in LECs

**Figure 4:**
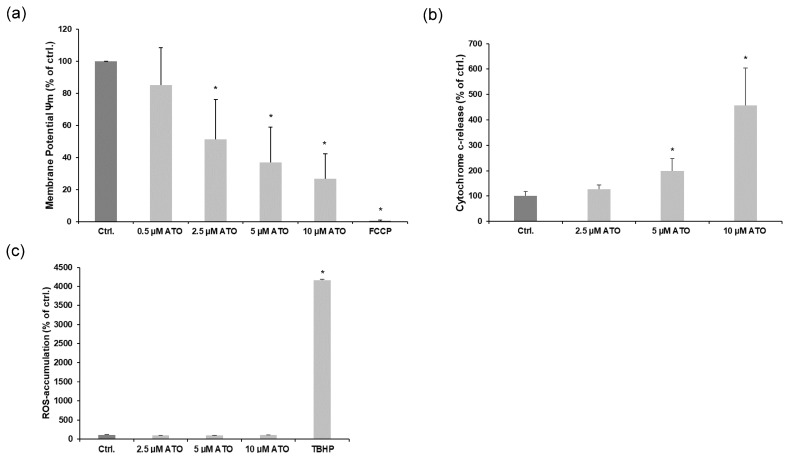
ATO triggers apoptosis through mitochondrial pathway. (**a**) Mitochondrial membrane potential assay was conducted using FACS with LECs treated with ATO (0.5–10 µM) or with 100 nM FCCP as a positive control for 24 h as indicated. Mean values from four independent experiments are shown as mean ± SD. We analyzed the data using Kruskal–Wallis test. * *p* < 0.05 vs. ctrl. (**b**) Cytosolic cytochrome c was quantified using an enzyme-linked immunosorbent assay. Mean values from four independent triplicate experiments are depicted ± SD (error bars); data are expressed as cytochrome release in percentage (%) with regard to solvent controls (=100%; H_2_O). Significance was determined by Kruskal–Wallis test with * *p* < 0.05 vs. ctrl. (**c**) ROS accumulation assay was performed with LECs treated with increasing concentrations of ATO (0.5–10 µM) or with 50 µM TBHP as a positive control for 24 h as indicated. Mean values from three independent triplicate experiments are shown as mean ± SD. Mean value of ctrl. was set to 100%. Mean values of remaining samples are shown as percentage of ctrl. Data were analyzed using Kruskal–Wallis test. * *p* < 0.05 vs. ctrl.

During intrinsic apoptosis, the membrane potential of mitochondria is decreased, which leads to the release of certain apoptosis triggering factors such as cytochrome c, which is followed by caspase 9 activation. To investigate whether increasing concentrations of ATO also lead to changes in mitochondrial membrane potential, an assay using FACS was conducted. We found that increasing concentrations of ATO (0.5–10 µM) decreased the mitochondrial membrane potential in LECs. The positive control (100 nM FCCP) abolished the membrane potential (Figure 4a). Consistent with this result, ATO induced cytochrome c release from mitochondria to cytosol in LECs in a concentration-dependent manner (Figure 4b). Next, we investigated the impact of ATO on intracellular ROS accumulation in LECs, which is an important step in mitochondrial membrane permeabilization and the subsequent release of cytochrome c. In our experiments, we observed that none of the used concentrations of ATO (2.5–10 µM) led to a significant difference in measured ROS activity in LECs, whereas the positive control (50 μM TBHP) led to an increase from 100% (ctrl. untreated) to around 4000% (Figure 4c). Taken together, our results demonstrate that ATO decreases the mitochondrial membrane potential with subsequent cytochrome c release independently of ROS induction in LECs.

### 3.4. Induction of Cell Death Markers by ATO Treatment

**Figure 5:**
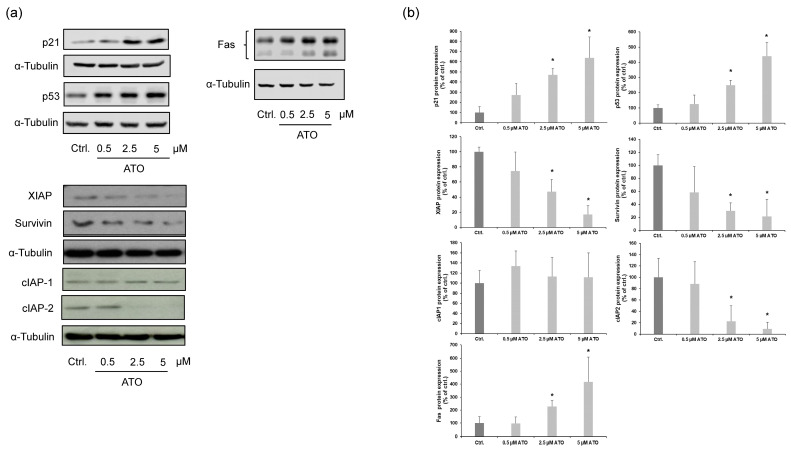
Induction of cell death markers by ATO treatment. (**a**) LECs were treated with 0.5, 2.5 and 5 µM ATO for 24 h as indicated. Apoptosis-related proteins were analyzed with Western blotting using the cell lysates. (**b**) Relative protein expression levels were semi-quantified by densitometric analysis. Data are expressed as the mean ± SD from at least four independent experiments. We analyzed the data using Kruskal–Wallis test. * *p* < 0.05 vs. ctrl.

Western blot analysis was performed to determine the expression of apoptosis-related markers that might be involved in the activation of ATO-induced intrinsic and extrinsic apoptotic pathways in LECs. In our experiments, we demonstrated that ATO upregulated the protein expression levels of p21 and p53 in a dose-dependent manner. Additionally, we observed that ATO reduced the expression of anti-apoptotic proteins survivin, XIAP and cIAP-2. Treating LECs with different concentrations of ATO did not lead to any significant differences in the expression pattern of cIAP-1. Furthermore, we found that ATO increased the protein expression of the important extrinsic apoptosis marker Fas (Figure 5a,b). These results demonstrate that ATO induces apoptosis in LECs by upregulating the tumor suppressor genes p21 and p53; downregulating the anti-apoptotic proteins survivin, XIAP and cIAP-2; and upregulating the extrinsic apoptosis-related protein Fas.

### 3.5. ATO Reduces the Expression of Important Endothelial Cell Receptors

**Figure 6:**
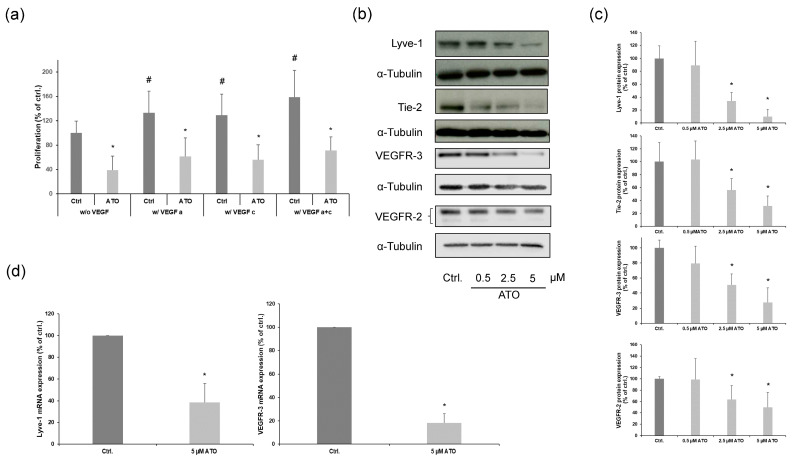
ATO reduces the expression of important endothelial cell receptors. (**a**) Cells were exposed to 5 µM ATO in the presence or absence of 20 ng/mL VEGF-A and 100 ng/mL VEGF-C for 24 h as indicated. Cell proliferation was analyzed by BrdU incorporation. The means ± SD of the eight independent experiments performed in triplicate are shown. Data were analyzed using Kruskal–Wallis test. * *p* < 0.05 vs. ctrl., ^#^ *p* < 0.05 vs ctrl. w/o VEGF. (**b**) Representative Western blotting analysis of important endothelial cell receptors in LECs treated with ATO (0.5–5 µM) for 24 h as indicated. (**c**) Relative protein expression levels were determined via densitometry and normalized against α-tubulin. Controls are set to 100. Data are expressed as the mean ± SD from at least five independent experiments. We analyzed the data using Kruskal–Wallis test. * *p* < 0.05 vs. ctrl. (**d**) Real-time PCR data of mRNA after treatment with 5 µM ATO for 24 h, showing the relative expression levels of VEGFR-3 and Lyve-1 and normalized against β-actin mRNA. Controls are set to 100. Data are expressed as the mean ± SD of the six independent experiments. Significance was determined by Mann–Whitney rank sum test with * *p* < 0.05 vs. ctrl.

Our results showed that the ability of ATO-treated LECs to build proper tubes was impaired significantly (Figure 1c). Therefore, we analyzed the impact of ATO on pro-angiogenic signaling. First, we demonstrated that ATO inhibited the VEGF-A and -C-induced proliferation of LECs, observing that ATO could modulate angiogenic signaling pathways (Figure 6a). Due to this, Western blot analysis with LECs treated with 0.5, 2.5 and 5 µM ATO as indicated for 24 h was conducted to examine the protein expression levels of important endothelial cell receptors that play major roles in triggering lymphangiogenesis. In our analysis, we found that ATO inhibited the expression of vascular endothelial growth factor receptor-2 and -3 (VEGFR-2/3), angiopoietin receptor Tie-2 and lymphatic vessel endothelial hyaluronan receptor Lyve-1 in a concentration-dependent manner (Figure 6b). Additionally, we found that 5 µM ATO reduced the mRNA expression levels of VEGFR-3 and Lyve-1 (Figure 6c). In summary, we demonstrate that the anti-lymphangiogenic effects of ATO are partly mediated by interfering with pro-survival signaling pathways through diminished endothelial VEGFR-3, Lyve-1 and Tie2 expression in LECs.

## 4. Discussion

Recently, studies have shown that lymphangiogenesis is an important step in tumor progression [18]. Lymph node involvement has long been recognized as a significant marker of poor prognosis in different types of tumors. Studies have revealed that intratumoral lymphatics and lymphatic invasion in melanoma and breast cancer are correlated with distant metastasis and reduced disease-free survival [24,25]. Furthermore, various studies have confirmed that increased lymphatic invasion in melanoma and breast cancer is associated with sentinel lymph node metastasis and serves as a prognostic marker for distant metastasis [26,27]. Taken together, it is assumed that therapies targeting tumor lymphangiogenesis might contribute to a reduction in malignant cancer progression [18]. In our study, we examined the effects of ATO on lymphangiogenesis in human lymphatic endothelial cells. ATO is a chemotherapy drug and exhibits low toxicity when used in the treatment of APL [2]. ATO has been shown to induce anti-lymphangiogenic activity in a mouse model of gastric cancer by various mechanisms, including the downregulation of pro-lymphangiogenic factors such as VEGFR-3 and VEGF-C in cancer cells [19]. In addition, it has been demonstrated that ATO impairs angiogenesis in vitro and in vivo [16,28,29]. Ge et al. demonstrated that ATO inhibited the proliferation and viability of human umbilical vein endothelial cells (HUVEC) and decreased their migration. Furthermore, they demonstrated that ATO reduced the expression of the pro-angiogenic factor VEGF-A through the induction of Ets-2 and miRNA-126 [30]. Additionally, Sun and colleagues demonstrated that ATO suppresses angiogenesis and induces autophagy in HUVECs through the upregulation of forkhead box protein O3 [28]. In the study of Zhang and colleagues, low dosages of ATO decreased the formation of vasculogenic mimicry (VM) in hepatoblastoma cells in vitro and in vivo via reducing the expression levels of VM-associated proteins such as VE-cadherin and MMP-9 [31]. Taken together, these findings suggest the growing importance of ATO and indicate that it could be an attractive candidate for anti-angiogenic therapy. To date, however, to the best of our knowledge, the anti-lymphangiogenic effects of ATO have not been studied on human lymphatic endothelial cells. In this study, the anti-lymphangiogenic effects of ATO in LECs were confirmed to be due to apoptosis and the inhibition of important endothelial cell receptors. To analyze the effects of ATO on LECs, LECs were treated with low-dose ATO (0.5–10 µM) for 24 h. Lymphatic endothelial cells showed a significant decrease in proliferation and tube formation starting from 2.5 µM. The cell cycle is important for the regulation of cell proliferation and duplication. We demonstrated that ATO induced subG0/G1 arrest, thus suggesting enhanced apoptosis. Although subG0/G1 arrest was accompanied by the induction of the cell cycle-regulating proteins p21 and p53, ATO treatment did not lead to visible cell cycle arrest in LECs, indicating a pro-apoptotic role of p21 and p53 for ATO-mediated apoptosis. In line with our findings, recent evidence has shown that ATO can induce apoptosis in a p21- and p53-dependent manner in other tumor models (e.g., hepatocellular carcinoma and glioma) [13,32]. Numerous studies have revealed that ATO can enhance the extrinsic apoptotic pathway by upregulating cell surface death receptors and/or ligands (e.g., Fas and TRAIL), leading to the activation of caspase 8 [33,34]. Furthermore, it has been found that ATO activates the intrinsic apoptotic pathway in different tumor cell lines through mitochondrial membrane disruption, resulting in the release of cytochrome c and the consecutive activation of caspases 3/7 and 9 [35,36]. In our study, we demonstrated that ATO activated both the intrinsic and extrinsic apoptotic pathways in human lymphatic endothelial cells by activating caspases 3, 8 and 9. The IAP and Bcl-2 proteins (such as cIAP-1/2, XIAP, survivin and Bcl-xL) are the key regulators of the intrinsic apoptosis pathway and cause mitochondrial disruption to release cytochrome c, which induces caspase activity and apoptosis [37]. Therefore, the expression pattern of these proteins in LECs after ATO treatment was verified. In our study, we demonstrated that ATO activated the intrinsic apoptotic pathway in human lymphatic endothelial cells by decreasing the mitochondrial membrane potential, increasing cytochrome c release and downregulating the anti-apoptotic proteins survivin, XIAP and cIAP-2. Intracellular ROS production is a frequent effect of chemotherapeutic agents such as ATO and is important in activating apoptotic pathways [38]. In our experiments, we could exclude any effects of ATO on intracellular ROS production in LECs, indicating that ROS is not involved in ATO-induced endothelial apoptosis. The Fas–FasL pathway has been considered as a prototypic inducer of the extrinsic apoptotic pathway in normal and neoplastic cells [37]. We found that caspase 8 activation was accompanied by the ATO-induced upregulation of Fas. These findings confirm that ATO-induced apoptosis may be at least one mechanism responsible for the anti-lymphangiogenic properties of ATO. In summary, our study demonstrated that ATO activates apoptosis in primary human lymphatic endothelial cells through inducing the intrinsic and extrinsic apoptotic pathways. It is well known that lymphangiogenesis in cancer is induced by tumor cells producing cytokines and growth factors like VEGF-A or C and angiopoietins, which bind to VEGF receptors-2 and -3 and Tie-2 at the surfaces of LECs located at quiescent lymphatic capillaries. This leads to the proliferation and formation of new lymphatic vessels towards the primary cancer cells in a concentration-based manner [39]. In addition, it has been found that signaling via Lyve-1, a lymphatic endothelial cell-specific hyaluronan receptor, promotes the proliferation and migration of lymphatic endothelial cells [40]. Interestingly, Xiao YF and colleagues showed that ATO delayed tumor growth, inhibited the microvessel density, downregulated VEGFR-1 and VEGFR-2 expression in gastric tumor xenografts and disturbed the stimulating effect of VEGF-A on the growth of SGC7901 gastric cancer cells [41]. In vivo studies further demonstrated that ATO could inhibit the expression of pro-lymphangiogenic VEGF-C and VEGFR-3 in a mouse model of gastric cancer. However, the impact of ATO on lymphatic endothelial cells has not been examined [19]. Here, we showed that ATO inhibited the proliferation of LECs and that these effects could not be abolished by the addition of the pro-lymphangiogenic growth factors VEGF-A and -C, indicating that ATO could modulate pro-lymphangiogenic signaling pathways. Since VEGF-A and -C bind to VEGFR-2 and -3, which leads to endothelial cell survival, proliferation and migration, we hypothesized that the suppression of these important endothelial receptors by ATO may represent an additional mechanism for ATO-mediated anti-lymphangiogenic effects. Furthermore, we examined the protein expression patterns of the important lymphatic endothelial cell receptors Tie-2 and Lyve-1. Our hypothesis was verified by our observation that ATO decreased the protein expression of VEGFR-2 and -3, Tie-2 and Lyve-1 in LECs. In addition, VEGFR-3 and Lyve-1 mRNA expression was decreased after ATO treatment. Due to the major role of these surface proteins in the process of lymphangiogenesis, particularly on the cell survival, proliferation, migration and sprouting of LECs, it can be assumed that these reduced levels led to the measured reduction in proliferation and impaired tube formation ability in LECs. Taken together, our results provide, for the first time, strong evidence that the ATO-related suppression of endothelial cell survival is partially mediated by the inhibition of specific lymphatic endothelial cell receptors.

## 5. Conclusions

Taken together, the results of our study present strong evidence that ATO demonstrates unique anti-lymphangiogenic effects in primary human lymphatic endothelial cells through the activation of both intrinsic and extrinsic apoptotic pathways. ATO-induced cell death was associated with a decrease in mitochondrial membrane potential; increased cytochrome c release; the downregulation of the anti-apoptotic proteins survivin, XIAP and cIAP-2; the upregulation of the pro-apoptotic proteins p21 and p53; and the induction of the expression of Fas. We also demonstrated for the first time that ATO reduced the expression of the important endothelial cell receptors VEGFR-2 and -3, Tie-2 and Lyve-1, demonstrating that ATO could influence specific pro-lymphangiogenic signaling pathways. Finally, our study has uncovered new targets in human lymphatic endothelial cells for the anti-lymphangiogenic effects of ATO, which may be an interesting therapeutic strategy in the treatment of tumor-associated lymphangiogenesis.

## Data Availability

All data supporting the findings in this study are included within the manuscript.
